# A Review on the Preparation of LDHs/Biochar Composites and Their Application in Water Pollution Control

**DOI:** 10.3390/ma19132867

**Published:** 2026-07-04

**Authors:** Yan Li, Nannan Guo, Letao Zhang, Chengwei Fan, Zhengqiang Ma, Ting Li, Xiaoyu Zhou

**Affiliations:** 1 Xinjiang Key Laboratory of New Energy Materials and Green Chemical Engineering, College of Chemistry and Environmental Engineering, Xinjiang Institute of Engineering, Urumqi 830091, China; zhangletao2004@163.com (L.Z.); fcw@xjie.edu.cn (C.F.); 15379130251@163.com (Z.M.); 18688534313@163.com (T.L.);; 2 State Key Laboratory of Chemistry and Utilization of Carbon Based Energy Resources, Xinjiang University, Urumqi 830017, China; guonan067@163.com

**Keywords:** layered double hydroxide, biochar, composite material, pollutants, water pollution control

## Abstract

This article systematically reviews the structural characteristics of layered double hydroxides and biochar (LDHs/biochar) composites, summarizes the features and optimization strategies of preparation methods such as coprecipitation, hydrothermal synthesis, ball milling, and calcination–reconstruction, analyzes their adsorption performance and mechanisms in controlling various water pollutants including organic contaminants, heavy metals, and nutrients, and provides insights into future research trends and practical applications, aiming to offer references for improving material performance and promoting practical use. The existing research results show that LDHs/biochar composites exhibit good application potential for various pollutants, such as dyes, antibiotics, heavy metal ions, and phosphates. The coprecipitation method is simple and easy to operate, and the LDHs/biochar composites prepared by this method exhibit favorable adsorption performance, with potential for industrial-scale production. The mechanisms of pollutant removal by LDHs/biochar composites primarily include electrostatic attraction, ion exchange, hydrogen bonding, complexation, and π–π electron interactions. Both the biomass type and the LDH type influence the adsorption performance of the composites. Therefore, designing LDHs/biochar composites based on pollutant characteristics and adsorption mechanisms is key to achieving effective pollution control. Currently, research on target pollutant-oriented material design and material regeneration remains underdeveloped and requires further breakthroughs.

## 1. Introduction

Driven by rapid industrialization, accelerating urbanization, and intensified agriculture, large volumes of wastewater containing heavy metal ions, anionic pollutants, and persistent organic pollutants are discharged into aquatic environments without adequate treatment. These pollutants exhibit high toxicity, resistance to biodegradation, and strong bioaccumulation potential, thereby posing threats to aquatic ecosystem security and human health. Accordingly, the development of efficient, cost-effective, and eco-friendly wastewater treatment technologies has emerged as a critical and urgent issue in environmental science. Among various water treatment strategies, adsorption is considered one of the most promising approaches owing to its simple operation, high removal efficiency, and low risk of secondary pollution [1,2,3].

In the course of exploring adsorbent materials, researchers have successively developed various single-component adsorbents. Activated carbon, featuring a well-developed pore structure and large specific surface area, was among the earliest widely adopted materials for the removal of organic pollutants and certain heavy metals from wastewater. Nevertheless, its high preparation cost, limited adsorption selectivity, and particularly unsatisfactory performance in targeting anionic contaminants have restricted its large-scale application in low-value wastewater treatment [4]. Subsequently, natural mineral materials such as zeolites and clays have attracted considerable attention owing to their abundant reserves and low cost. Zeolites exhibit unique sieving effects and excellent cation exchange capacity, while clay minerals occupy an important position in adsorption due to their layered structures and surface reactivity. However, unmodified natural minerals generally suffer from limited adsorption capacity and poor selectivity toward specific pollutants, particularly anionic contaminants [5,6]. To address these shortcomings, researchers have fabricated a series of modified adsorbents through acid-base modification, metal oxide loading, or organic functional group decoration. Although such modifications have improved performance to some extent, the preparation procedures are often complex and may introduce new environmental risks.

Layered double hydroxides, as typical anionic layered materials, exhibit advantages in adsorption applications due to their unique structural features. LDHs are composed of positively charged host layers and exchangeable interlayer anions, and possess memory effects, tunable metal compositions, and satisfactory anion exchange capacity. They exhibit high adsorption affinity for anionic contaminants including phosphate and arsenate [7,8]. However, pure LDH materials face two major bottlenecks in practical applications: first, their powdery nature tends to aggregate in aqueous solution, resulting in a decrease in active sites; second, their poor mechanical strength makes efficient solid–liquid separation and recycling difficult, which hinders their engineering application [9,10].

As single materials struggle to meet the integrated requirements of high capacity, low cost, easy separation, and environmental friendliness, the fabrication of composites with synergistic effects by combining two or more functional materials has become a research hotspot in the field of environmental functional materials. Against this background, the introduction of biochar provides a new strategy to address the limitations of LDHs. Biochar is a carbon-rich material produced by the pyrolysis of biomass under limited-oxygen conditions. With a wide range of feedstocks, it features a low-cost, well-developed pore structure, and abundant oxygen-containing functional groups on the surface. While adsorbing heavy metals and organic pollutants, biochar also enables the resource utilization of waste biomass, which conforms to the green environmental concept of “treating waste with waste” [11,12].

The LDHs/biochar composite fabricated by combining LDHs and biochar integrates the structural advantages of the two components. On the one hand, biochar acts as a porous scaffold that can effectively anchor and disperse LDH particles, preventing their agglomeration and thus exposing more active sites. On the other hand, the LDHs loaded onto the biochar surface endow the composite with anion-exchange performance and abundant surface hydroxyl groups, strengthening the specific adsorption toward anionic pollutants [13,14,15].

Currently, LDHs/biochar composites have been widely employed in the advanced treatment of various types of wastewater, including the resource recovery of phosphorus-containing wastewater, the purification of water bodies co-contaminated with heavy metals, and the catalytic degradation of organic wastewater [16,17,18,19]. Although numerous studies have demonstrated their effectiveness, systematic reviews remain insufficient on several key aspects: the relationship between microstructure and adsorption performance, the influence of different preparation methods on their microstructure, the mechanisms of interaction with different pollutants, and their long-term stability and regeneration performance under complex real-world water conditions.

Against this background, this paper aims to systematically review the research progress of LDHs/biochar composites. First, the basic structural characteristics of LDHs and biochar, as well as the adsorption mechanism of the composites, are introduced. Next, the current mainstream preparation methods and their effects on material properties are summarized. Subsequently, this review highlights the adsorption mechanisms and application performance of such composites for heavy metals, anionic nutrients, and organic pollutants in water treatment. Finally, future research directions in this field are prospected, with a view to providing a theoretical basis and technical reference for the development of novel high-efficiency adsorbents and their practical application in water pollution control.

## 2. Structure and Properties of LDHs/Biochar Composites

Layered double hydroxides are a class of hydrotalcite and hydrotalcite-like compounds composed of positively charged brucite-like layers and exchangeable interlayer anions. The positively charged layers consist of divalent or trivalent metal cations octahedrally coordinated with hydroxide ions [20,21]. Common layer cations include Mg^2+^, Zn^2+^, Ni^2+^, Al^3+^ and Fe^3+^, while typical interlayer anions are NO_3_^−^, CO_3_^2−^ and Cl^−^.

LDHs exhibit a distinctive combination of structural and surface properties. Their permanently positive surface charge endows them with high anion exchange capacity and hydrophilicity. In addition, the abundant surface hydroxyl groups and large specific surface area provide ample active sites for pollutant capture, while their thermal stability and structural reconstructability (memory effect) facilitate regeneration and reuse [22].

**Figure 1:**
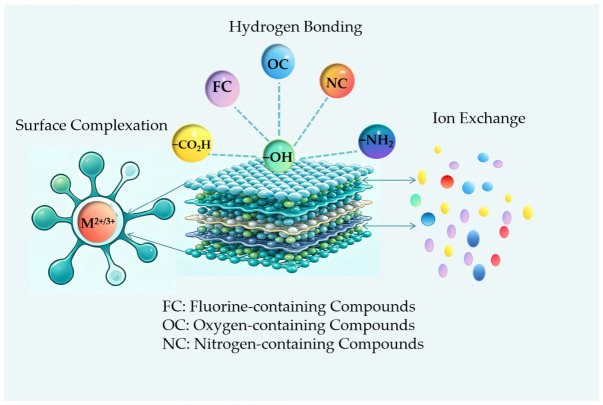
Schematic diagram of the adsorption mechanism of LDHs.

Through multiple molecular interactions including ion exchange, metal ion complexation, and hydrogen bonding (Figure 1), LDHs have shown promising adsorption capacities for various waterborne pollutants [23]. However, a major obstacle to their practical application is the inherent tendency of LDH particles to aggregate in aqueous solution, which reduces the availability of active sites and hinders large-scale implementation [24,25,26].

To address this limitation, researchers have explored suitable support materials to immobilize and disperse LDH particles. Among these, biochar has attracted considerable attention as a suitable candidate. Biochar is a porous carbon-rich material produced by the pyrolysis and carbonization of biomass feedstocks under limited-oxygen conditions [27]. It features a highly developed hierarchical pore structure encompassing micropores, mesopores, and macropores, with specific surface areas reaching up to 1000 m^2^/g.

The surface of biochar is typically rich in oxygen-containing functional groups, such as hydroxyl (-OH), carboxyl (-COOH), and phenolic hydroxyl (Ar-OH) groups. The composition and content of these surface functional groups can be precisely regulated through modification treatments or by adjusting pyrolysis conditions, offering flexibility in tailoring adsorption properties [28,29].

**Figure 2:**
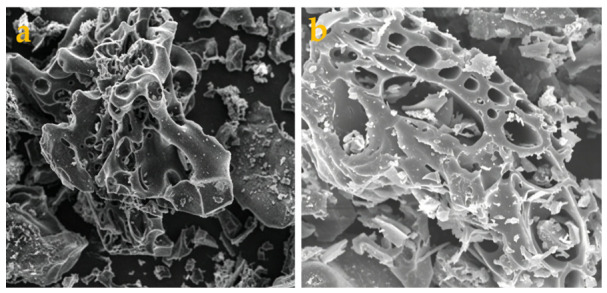
Biochars derived from different biomasses ((**a**) wheat grain biochar [31], (**b**) rice husk bio-char [32]).

Owing to these microstructural characteristics, biochar demonstrates effective sequestration capacities for heavy metals, organic pollutants, and other contaminants. Notably, biochars derived from different biomass precursors exhibit considerable variations in microstructure and adsorption behavior, as shown in Figure 2. It should be noted, however, that biochar generally carries a negatively charged surface under neutral to alkaline conditions, which limits its affinity for anionic pollutants due to electrostatic repulsion [30].

**Figure 3:**
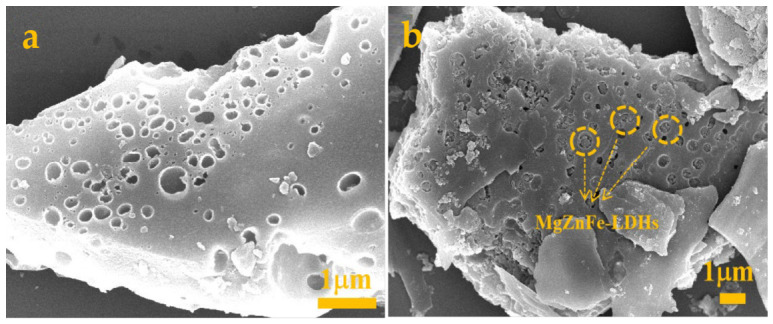
SEM images of biochar (**a**) and its composite with MgZnFe-LDHs (**b**) [33].

The LDHs/biochar composite integrates the complementary structural merits of both components. On the one hand, biochar provides a well-developed three-dimensional porous scaffold with a wide pore size distribution. SEM observations reveal that macropores dominate this structure, accompanied by a hierarchical “pore-in-pore” architecture in which smaller pores and mesopores are interconnected. On the other hand, LDH nanosheets can be uniformly loaded onto the biochar surface and infiltrate into the macroporous channels during composite fabrication. This loading renders the surface morphology rougher and, through the partitioning effect of LDH sheets, generates additional small pores, leading to enhanced overall porosity, as illustrated in Figure 3. Importantly, the positively charged LDH layers effectively compensate for the limited anion affinity of pristine biochar, enabling the composite to capture both cationic and anionic pollutants simultaneously.

Studies [34,35] have demonstrated that most LDHs-modified biochars possess elevated specific surface area (SSA) and total pore volume (TPV), which contribute to improved adsorption capacity toward target pollutants. The underlying mechanism is twofold: the biochar matrix with its large surface area provides space for LDH loading, while the enlarged SSA facilitates physical adsorption and the newly introduced surface functional groups enhance chemisorption. As presented in Samples 1–8 of Table 1, pine cone biochar loaded with MnFe-LDHs and coconut fiber biochar loaded with ZnAl-LDHs achieve increases in both specific surface area and adsorption capacity.

However, it has also been reported that LDH nanosheets may partially block biochar pores during composite fabrication, leading to a decline in specific surface area [36]. Despite this, the abundant active sites generated through modification endow the resulting composite with higher adsorption capacity than pristine biochar. This is exemplified by Samples 9–11 in Table 1: despite a reduced surface area, the AlMg-LDHs/biochar composite delivers a Pb(II) adsorption capacity of 294.0 mg/g, surpassing both pristine coconut shell biochar and pure AlMg-LDHs. This enhancement is primarily attributed to the increased density of oxygen-containing functional groups—the combined content of C–O–C and O=C–O groups in Sample 11 is 14.5% higher than that in Sample 9. Similar findings have been reported in references [11,37].

As shown by Samples 12–20 in Table 1, the adsorption performance of LDHs/biochar composites is governed by three key factors: the metal cation composition of LDH layers, the pyrolysis temperature of biochar, and the type of biomass feedstock [15,30,38,39,40]. The ionic composition of the LDH layers determines their surface charge density, which in turn influences electrostatic interactions and interlayer ion exchange capacity. The pyrolysis temperature not only regulates the pore structure of biochar but also alters the abundance of surface functional groups; an appropriate temperature develops well-defined porosity while preventing pore collapse. Meanwhile, the choice of biomass feedstock affects the specific surface area and surface functional groups of the resulting biochar, thereby modulating the contributions of both physical and chemical adsorption.

In addition to material-intrinsic parameters, synthesis conditions and operational adsorption parameters also exert influence on adsorption performance. Therefore, systematic investigation and comprehensive evaluation are essential before scaling up these composites for practical applications.

**Table 1:** Physical Properties and Adsorption Capacity of LDHs/biochar Composites.

No	LPI	BRM	TP	SSA (m^2^/g)	APD (nm)	TPV (cm^3^/g)	AC (mg/g)	References
1	-	Pine Cone	Crystal Violet	43.41	-	0.037	13.477	[34]
2	Mn-Fe	Pine Cone	Crystal Violet	98.85	-	0.19	41.152	[34]
3	-	Coconut Fiber	Pb(II)	8.29 (500 °C)	3.82	0.01	109.75	[35]
4	-	Coconut Fiber	Pb(II)	20.54 (600 °C)	5.64	0.02	109.05	[35]
5	-	Coconut Fiber	Pb(II)	180.49 (700 °C)	2.18	0.23	132.27	[35]
6	Zn-Al	Coconut Fiber	Pb(II)	121.28 (500 °C)	8.10	0.11	132.45	[35]
7	Zn-Al	Coconut Fiber	Pb(II)	180.49 (600 °C)	5.69	0.16	134.74	[35]
8	Zn-Al	Coconut Fiber	Pb(II)	492.21 (700 °C)	5.29	0.26	163.89	[35]
9	-	Coconut Shell	Pb(II)	1195	5.10	0.888	13.3	[36]
10	Mg-Al	-	Pb(II)	-	-	-	126.0	[36]
11	Mg-Al	Coconut Shell	Pb(II)	394.8	10.40	0.725	294.0	[36]
12	Fe-Mg	Bagasse	Pb(II)	147.26	13.88	-	402.70	[38]
13	Fe-Mg	Bagasse	Cu(II)	147.26	13.88	-	68.50	[38]
14	Fe-Mg	Bagasse	As(V)	147.26	13.88	-	21.68	[38]
15	Al-Mg	Hickory-chip	Congo Red	223.5	3.1	0.199	1045.6	[39]
16	Al-Mg	Peanut-shell	Congo Red	216.4	3.3	0.195	1195.0	[39]
17	Mg-Al	Macadamia Nut Shell	Phosphate	378	-	-	43.05	[15]
18	Mg–Al	Bamboo	Phosphate	10.1	-	-	172	[30]
19	Ca-Mg-Al	Bamboo	As(III)	229.28	1.6835	0.0965	206.59	[40]
20	Ca-Mg-Al	Bamboo	Cd(II)	229.28	1.6835	0.0965	216.18	[40]

## 3. Preparation Methods of LDHs/Biochar

**Figure 4:**
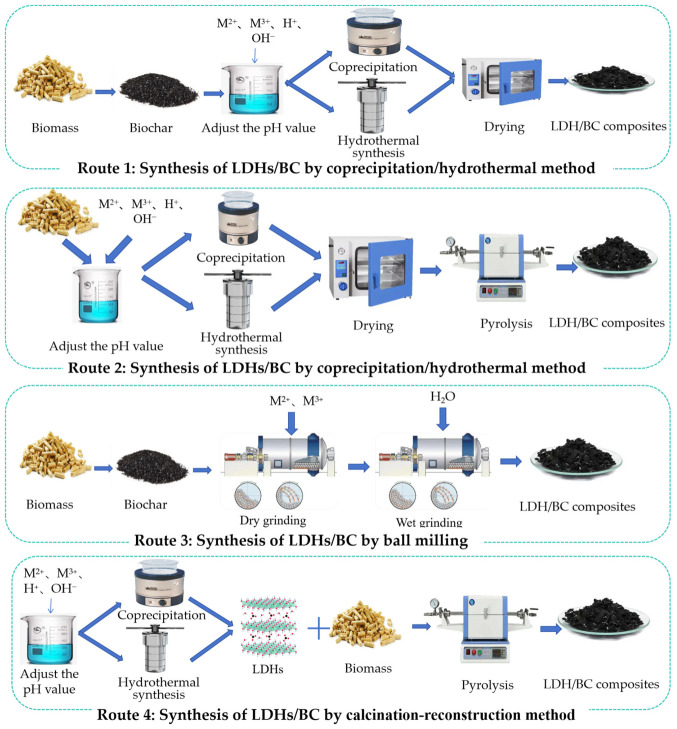
Preparation methods of LDHs/biochar composites.

The synthesis methods of LDHs/biochar composites are derived from the traditional preparation processes of LDHs. Among the mainstream LDH preparation techniques—including coprecipitation, hydrothermal synthesis, urea hydrolysis, the sol-gel method, and mechanical grinding—coprecipitation and hydrothermal synthesis are widely used for the controllable fabrication of LDHs/biochar composites. A schematic illustration of the typical preparation routes is presented in Figure 4.

The coprecipitation method can be implemented via two strategies. The first involves the synthesis of biochar first, followed by a coprecipitation reaction with metal ions at a predetermined ratio to directly form the composite. The second entails mixing biomass feedstocks with metal salts, followed by coprecipitation and subsequent pyrolysis under limited-oxygen conditions. The hydrothermal method can be subdivided into three approaches: two of them share a similar concept with the coprecipitation strategies but replace the coprecipitation step with a hydrothermal reaction; the third approach is a two-stage process in which LDHs are first synthesized independently, then thoroughly mixed with biomass feedstocks, and finally subjected to pyrolysis—this variant is also referred to as the calcination–reconstruction method.

Compared with hydrothermal synthesis, coprecipitation offers the advantage of mild reaction conditions. Theiss et al. [41] noted that this method features simple operation and low cost, exhibiting potential for industrial scale-up. Similarly, Sun et al. [42] concluded that coprecipitation is a classic approach for preparing LDHs/biochar composites, capable of yielding products of high quality. In addition to these two mainstream methods, several studies have explored the fabrication of LDHs/biochar composites via ball milling; however, the resulting adsorption performance is generally inferior to that achieved by coprecipitation and hydrothermal routes. The following sections provide a detailed discussion of each preparation method.

### 3.1. Coprecipitation Method

As noted above, coprecipitation encompasses two main pathways: coprecipitation of biomass feedstocks with metal salts followed by anaerobic pyrolysis, and coprecipitation using pre-prepared biochar with metal salts. The latter approach has been more widely adopted owing to its greater controllability over biochar properties.

A representative example is the work of Xiao et al. [22], who fabricated Fe_3_O_4_-MgAl-LDHs/biochar composites using reed straw biochar as the matrix. In their procedure, reed powder was first impregnated with iron salts, followed by coprecipitation with magnesium and aluminum nitrates and subsequent anaerobic pyrolysis in a muffle furnace. The resulting composite achieved a phosphorus removal rate of 99.24% from an initial concentration of 20 mg/L. Employing a similar approach, Tan et al. [43,44,45] prepared LDHs/biochar composites and demonstrated their effectiveness in the adsorptive removal of crystal violet, cadmium, and phosphate. In another study, El-Sawaf et al. [46] synthesized multifunctional MgAl-LDH/biochar composites through in situ coprecipitation using KOH-chemically activated biochar with Mg–Al salts. The product possessed a high specific surface area of 712 m^2^/g and delivered adsorption capacities of 243.5 mg/g for tetracycline and 198.7 mg/g for stearic acid, respectively.

Collectively, these studies illustrate the versatility of the coprecipitation method in producing LDHs/biochar composites with high loading uniformity and favorable adsorption performance across a range of target pollutants.

### 3.2. Hydrothermal Synthesis Method

The hydrothermal method is a simple and environmentally friendly synthetic approach widely applied in the preparation of composites and ceramic materials. In a typical procedure, a metal ion solution of predetermined composition is uniformly mixed with biochar, and the composite is obtained after hydrothermal reaction.

Chen et al. [47] prepared LDHs/biochar composites using this method. They first activated *camellia oleifera* shell with phosphoric acid and pyrolyzed it at 500 °C to obtain biochar. The biochar was then mixed with Ni/Al, Mg/Al, or Ni/Mg/Al salt solutions, dispersed under ultrasonic irradiation, and transferred into a PTFE-lined autoclave for hydrothermal reaction. The resulting composite exhibited high adsorption capacities of 271.5 mg/g for Cr(VI) and 412.8 mg/g for methyl orange. In a binary pollutant system, the removal efficiencies of both pollutants were simultaneously enhanced—a synergistic effect possibly arising from complementary adsorption sites or cooperative binding mechanisms.

Fu et al. [48] employed a different strategy, first preparing magnetic biochar through impregnation combined with pyrolysis and then loading MgFe-LDHs onto it via the hydrothermal method. The resulting magnetic composite exhibited maximum adsorption capacities of 213.156 mg/g for Cd^2+^ and 43.291 mg/g for Ni^2+^, respectively.

### 3.3. Ball Milling Method

The ball milling method for LDHs/biochar composite preparation is a two-step process consisting of dry milling and wet milling. In the dry milling stage, biochar, metal salts for LDH synthesis, and alkali are introduced into a ball mill to achieve uniform mixing of raw materials. In the subsequent wet milling stage, an appropriate amount of distilled water, necessary for LDH formation, is added to the mixture, followed by continuous grinding to obtain the final product.

Zhou et al. [49] compared the phosphorus recovery performance of CaAl-LDH/biochar composites prepared by ball milling, hydrothermal, and precipitation methods. Among the three, the precipitation-derived composite exhibited the highest adsorption capacity (76.51 mg/g), followed by the hydrothermal method (66.44 mg/g) and the ball milling method (60.78 mg/g). These results indicate that while ball milling offers the advantage of solvent-free or solvent-minimized processing, the adsorption performance of the resulting composites is slightly inferior to that of materials prepared by wet-chemical routes.

### 3.4. Calcination–Reconstruction Method

The calcination–reconstruction method is founded on the “memory effect” of LDHs. Unlike coprecipitation and hydrothermal synthesis, this protocol requires the pre-synthesis of LDHs, which are then loaded onto the surface of biomass substrates. The LDH-loaded biomass is subsequently subjected to high-temperature pyrolysis under limited-oxygen conditions to produce LDOs/biochar composites. During pyrolysis, LDHs undergo dehydration, and the metal hydroxide layers transform into mixed metal oxides.

When the resulting LDOs/biochar composite is exposed to aqueous environments containing anionic pollutants, it not only adsorbs the target anions but also undergoes hydration-driven reconstruction of the original layered LDH structure. This reversible transformation is of significant value for material regeneration and recycling, particularly in the remediation of organic pollution [50].

## 4. Application of LDHs/Biochar in Water Pollution Control

### 4.1. Removal of Organic Pollutants

LDHs/biochar composites exhibit potential for organic pollutant removal, benefiting from the synergistic structural and chemical properties of both components. This section reviews representative studies on the adsorption of organic contaminants, covering structural advantages, key performance data, regeneration capability, and underlying mechanisms. A structural feature of these composites is the spatial segmentation effect exerted by LDH nanosheets on the macroporous biochar matrix. Such structural regulation endows the composite with a higher specific surface area compared with pristine biochar and pure LDHs, thereby enhancing the overall adsorption capacity.

A series of studies have demonstrated this structural advantage through different composite designs. For instance, a CoFe_2_O_4_/MnFe LDH/biochar composite was fabricated by modifying pine cone-derived biochar with CoFe_2_O_4_ magnetic nanoparticles and Mn–Fe layered double hydroxides [34]. The specific surface area increased progressively from 43.41 m^2^/g (raw biochar) to 98.85 m^2^/g after LDH incorporation, indicating that the introduction of LDH further modified the pore structure. Under optimal adsorption conditions, the composite achieved a crystal violet removal efficiency of up to 98.54%.

In another study, Mg–Al LDH/biochar composites were synthesized via coprecipitation using cattle bone as the biomass feedstock and applied to the adsorptive removal of caffeine and sodium diclofenac [51,52]. Adsorption equilibrium was rapidly attained within 20 min. Isotherm and thermodynamic analyses revealed that the adsorption process was exothermic, with adsorption capacity decreasing as temperature increased. At 40 °C, the maximum caffeine adsorption capacity reached 26.219 mg/g. For sodium diclofenac, the composite exhibited a removal rate of nearly 80% and an adsorption capacity of 5.96 mg/g—higher than raw biochar (2.35 mg/g). Importantly, the saturated adsorbent could be regenerated using methanol or a 0.5 mol/L NaCl solution and maintained favorable adsorption performance after three regeneration cycles, highlighting its reusability and practical feasibility.

The properties of the biochar matrix also play a key role in adsorption performance, even when the LDH composition is identical. Using sugarcane bagasse biomass, a CLDHs/biochar composite (calcined Mg–Al LDH/biochar) was prepared through a one-pot slow pyrolysis method [53]. Characterization confirmed that calcined Mg–Al LDH was uniformly coated onto the biochar surface during pyrolysis. The resulting composite delivered a tetracycline adsorption capacity more than twice that of pristine biochar, reaching a maximum of 1118.12 mg/g at 318 K.

An advantage of LDHs/biochar composites is their dual functionality toward both cationic and anionic organic pollutants. Mouhri et al. [54] developed a Cu_2_(OH)_2_CO_3_-CuAl LDH/biochar composite that enhances structural stability and adsorption performance. This material exhibited considerable removal efficiencies for both anionic Chrome Black T (94.43 mg/g) and cationic methylene blue (12.57 mg/g), demonstrating the versatility of the composite in treating cationic and anionic organic pollutants.

To elucidate the adsorption behavior observed in the above studies, mechanistic analyses have been conducted, revealing that the LDH component and the biochar component contribute through distinct yet complementary pathways. The LDH component provides anion exchange, hydrogen bonding, and metal ion complexation capabilities, while the biochar component, rich in surface functional groups, participates in hydrogen bonding and π–π interactions. Specifically, the aromatic rings within the biochar matrix can interact with the π–electron systems of organic pollutants through π–π stacking and p–π interactions [46,55].

**Figure 5:**
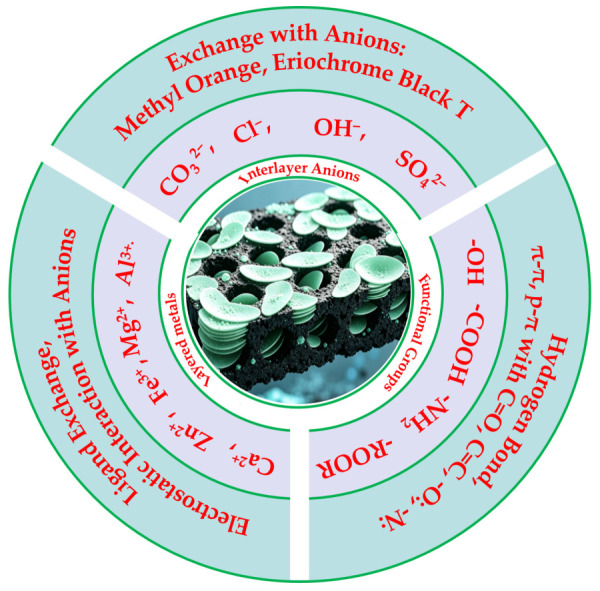
Adsorption Mechanism of Cationic Dyes by LDHs/Biochar.

Taking the adsorption of anionic Chrome Black T as an illustrative example, the mechanism involves multiple concurrent pathways (Figure 5). Ion exchange occurs between the pollutant and the interlayer anions (CO_3_^2−^ and Cl^−^) of LDHs, accompanied by electrostatic and coordination interactions with the layered Cu^2+^ and Al^3+^ cations. Simultaneously, the –N=N–, –NO_2_, and aromatic π–bond systems in Chrome Black T engage in π–π and p–π interactions with the –C=O, –COOH, –NH_2_ groups, and aromatic rings of the composite. Hydrogen bonds form between the N, O, and –OH groups of the dye and the –COOH, –NH_2_ functional groups, as well as N- and O-containing active sites on the composite surface. Ligand exchange further contributes to the overall adsorption process [53,54].

By contrast, the adsorption of cationic methylene blue is governed by two main pathways. Weak electrostatic interactions between methylene blue and the interlayer anions of LDHs serve as an auxiliary effect. The dominant contribution, however, arises from the abundant –OH groups on the material surface and the π electrons of the biochar aromatic rings, which enrich methylene blue molecules through hydrogen bonding and π–π electron attraction. This complementary dual mechanism—wherein LDHs primarily capture anions through ion exchange and complexation, while biochar predominantly binds cations and neutral organics via π–π interactions and hydrogen bonding—constitutes the foundation for the wide-ranging organic pollutant removal capability of LDHs/biochar composites.

### 4.2. Removal of Phosphate from Eutrophic Water

The massive discharge of industrial, agricultural, and domestic sewage has led to increasing water eutrophication. As a key contributor to this environmental problem, phosphate poses significant hazards to aquatic ecosystems and has attracted considerable research attention [56,57,58]. LDHs/biochar composites have emerged as a research hotspot for the adsorptive removal of phosphate, with studies focusing on the influences of LDH metal composition, metal ion molar ratio, and solution chemistry on adsorption performance and mechanisms [59,60]. This section reviews key findings in these aspects.

#### 4.2.1. Effect of Metal Ions in LDH Layers

The metal composition of LDH layers exerts a decisive influence on phosphate adsorption capacity. As shown in Table 2 (Rows 1–6), composites with different layer metal components exhibit clear differences in performance, even when synthesis routes and adsorption conditions are held constant. According to the literature [61], CaLa-LDH/biochar delivers the highest phosphate adsorption capacity of 100 mg/g under identical conditions, higher than MgAl-LDH/biochar, MgLa-LDH/biochar, MgFe-LDH/biochar, CaAl-LDH/biochar, and CaFe-LDH/biochar. This performance is attributed to the strong intrinsic affinity of lanthanum for phosphate ions [62]. Consistent findings have been reported in the literature [63]: the phosphate adsorption capacities of ZnFe-LDH/biochar, MgFe-LDH/biochar, ZnAl-LDH/biochar, and MgAl-LDH/biochar vary with changes in layer metal composition. Although a positive correlation between specific surface area and adsorption capacity is observed for most composites, the case of CaAl-LDH confirms that surface area is not the dominant factor governing performance. Rather, the specific chemical interactions of the metal species—particularly the strong affinity of Ca^2+^ and La^3+^ for phosphate—serve as the primary determinants of the adsorption process [64,65].

#### 4.2.2. Effect of Metal Ion Molar Ratio and Preparation Methods

Beyond the type of metal ions, the molar ratio of divalent to trivalent metals in the LDH layers also exerts an important influence on phosphate adsorption. Jiang et al. [66] synthesized ZnAl-LDH via a hydrothermal method and loaded it onto banana straw biochar, followed by calcination at 500 °C. The resulting composite self-assembled into microspheres with a typical hexagonal lamellar structure and exhibited thermal stability. Its phosphate adsorption behavior followed the Langmuir model, yielding a theoretical maximum adsorption capacity of 185.19 mg/g—a value remarkably higher than those of composites prepared with conventional metal ratios [61,63]. A key distinguishing factor was the Zn/Al molar ratio of 10, which exceeds the typical range of 2–4 reported in previous studies [68,69]. Whether this unusually high ratio can be reliably reproduced and whether it translates into genuine cost-effectiveness advantages warrant further systematic investigation.

In a different approach, Zhou et al. [67] loaded MgAl-LDH with a Mg/Al molar ratio of 4 onto corn straw biochar via the calcination–reconstruction method, achieving a theoretical maximum adsorption capacity of 54.932 mg/g. Compared with composites prepared by the coprecipitation method, this value exceeded that of a composite with a Mg/Al ratio of 3 [61] but fell below that of a composite with a Mg/Al ratio of 2 [63]. This non-monotonic trend suggests that multiple factors are at play. First, the biomass feedstocks differ among these studies, introducing variability in the biochar matrix properties. Second, the calcination step inherent to the calcination–reconstruction process removes interlayer anions (such as hydroxide and carbonate), thereby facilitating phosphate uptake during the reconstruction phase in aqueous solution. Third, the optimal Mg/Al ratio may depend on the specific combination of preparation method and biomass precursor, and further systematic comparisons under controlled conditions are needed to decouple these variables.

#### 4.2.3. pH-Dependent Adsorption Mechanisms

Solution pH is a critical parameter governing phosphate adsorption, as it influences both the speciation of phosphate and the surface charge of the adsorbent. Bolbol et al. [70] systematically investigated the effects of phosphate concentration, pH, and contact time on the adsorption performance of Fe/Mg-LDH/biochar composites and confirmed a pH dependence.

When the solution pH is below the point of zero charge (pHpzc), surface functional groups such as -OH and -COOH on the composite become protonated, carrying positive charges (-OH_2_^+^, -COOH_2_^+^). Under these acidic conditions, electrostatic adsorption dominates: the protonated surface groups attract anionic phosphate species (predominantly HPO_4_^2−^ and H_2_PO_4_^−^) through electrostatic interactions, as described in Equations (1)–(6) [58,71,72,73]. Biochar composites prepared by low-temperature pyrolysis (≤500 °C), which are rich in oxygen-containing functional groups, are particularly suitable for treating acidic phosphorus-containing wastewater from industrial, agricultural, and mining sources [37].

-OH + H^+^ → -OH_2_^+^ (1)

-COOH + H^+^ → -COOH_2_^+^ (2)

-OH_2_^+^ + HPO_4_^2−^ → -OH_2_^+^ − HPO_4_^2−^ (3)

-OH_2_^+^ + H_2_PO_4_^−^ → -OH_2_^+^ − H_2_PO_4_^−^ (4)

-COOH_2_^+^ + HPO_4_^2−^ → -COOH_2_^+^ − HPO_4_^2−^ (5)

-COOH_2_^+^ + H_2_PO_4_^−^ → -COOH_2_^+^ − H_2_PO_4_^−^ (6)

When the solution pH exceeds the pHpzc or the environment is alkaline, phosphate predominantly exists as PO_4_^3−^. Under these conditions, a different mechanism prevails: PO_4_^3−^ reacts with metal ions leached from the LDH lamellar structure to form insoluble precipitates, as shown in Equations (7) and (8) [73,74,75]. This precipitation reaction is irreversible and yields a high chemical adsorption capacity, making the material suited for the immobilization treatment of phosphorus in livestock breeding wastewater and domestic sewage [76].

M^2+^ + PO_4_^3−^ → M_3_(PO_4_)_2_↓ (7)

M^3+^ + PO_4_^3−^ → MPO_4_↓ (8)

In addition to electrostatic attraction and precipitation, other chemical adsorption processes—including ion exchange, surface complexation, and ligand exchange—also contribute to phosphate removal by LDHs/biochar composites [62,77,78,79]. Specifically, interlayer anions of LDHs (e.g., CO_3_^2−^, Cl^−^) can be exchanged with phosphate ions in solution; surface hydroxyl groups on both LDHs and biochar can form inner-sphere complexes with phosphate via ligand exchange; and metal cations in the LDH layers can directly coordinate with phosphate through surface complexation. These multiple concurrent mechanisms, operating synergistically across different pH ranges, underpin the efficiency and applicability of LDHs/biochar composites for phosphate removal from diverse water matrices.

### 4.3. Adsorption of Heavy Metal Ions from Wastewater

The LDHs/biochar composites have demonstrated potential for the removal of heavy metal ions from wastewater, with higher performance than both pristine biochar and pure LDHs in terms of adsorption capacity and selectivity [38,80,81,82,83,84,85]. This section reviews representative studies on the adsorption of Pb^2+^, Cu^2+^, and Cd^2+^, followed by a comparative discussion of the key factors governing adsorption performance, including biomass feedstock type, LDH metal composition, and preparation method.

Lead removal has been a focus in the development of LDHs/biochar adsorbents. Yuan et al. [86] used *Camellia oleifera* shells as the raw material to prepare magnetic biochar via a hydrothermal method. Then, they compounded the magnetic biochar with Zn/Al layered double hydroxide through a hydrothermal synthesis method to obtain a Zn/Al LDH magnetic biochar composite (LMB). The adsorption mechanism of LMB for Pb^2+^ was investigated through thermodynamic and kinetic studies, and the results showed that the adsorption reached equilibrium within 120 min. The initial concentration of the solution had an impact on the adsorption of Pb^2+^ by LMB, with an equilibrium adsorption capacity of 172.5 mg/g. Increasing the pH enhanced Pb^2+^ adsorption. The adsorption process of Pb^2+^ by LMB conformed to the Langmuir model, indicating monolayer adsorption that was primarily chemical in nature. The maximum adsorption capacity derived from the Langmuir model was 213.0 mg/g, and the adsorption capacity was higher than that of pure biochar and magnetic biochar.

Compared with the Zn/Al-LDH magnetic biochar reported above, the magnesium-iron layered double hydroxide magnetic biochar composite synthesized by Jia et al. [87] had a higher adsorption capacity for Pb^2+^. The composite interacted with Pb^2+^ via interlayer anions and surface hydroxyl groups, with a maximum adsorption capacity of 476.25 mg/g for Pb^2+^ at 25 °C. Kinetic and thermodynamic studies indicated that the adsorption process was a spontaneous endothermic reaction and was controlled by chemisorption.

Wang et al. [88] prepared a composite material consisting of biochar and manganese-aluminum layered double hydroxide, which can remove Cu(II) from aqueous solutions. Key factors affecting adsorption efficiency included adsorbent dosage, solution pH, and coexisting cations. The study showed that the Cu(II) adsorption process involved the formation of surface-precipitated Cu_2.5_(OH)_3_SO_4_ and the isomorphous substitution between Cu(II) and Mn(II). The Langmuir model yielded a maximum adsorption capacity of 74.07 mg/g for Cu(II), which was superior to that of other biochar-based adsorbents.

Tan et al. [44] loaded magnesium-iron layered double hydroxides onto kiwifruit branch biochar (KB) via liquid phase deposition to prepare a functionalized composite (KB/LDH). The effects of pH value, ionic strength, and coexisting ions on the adsorption performance were systematically investigated. The results indicated that the adsorption capacity of KB/LDH for Cd(II) was 25.6 mg/g, which was 14.4 times higher than that of the original biochar (1.78 mg/g). The adsorption behavior was affected by environmental factors: a lower ionic strength led to a higher Cd(II) adsorption capacity, and higher pH enhanced Cd(II) removal. The composite showed good resistance to interference from coexisting ions such as K^+^, Cl^−^, and NO_3_^−^. Mechanism analysis revealed that the coordination of hydroxyl groups on the LDH layers, the complexation of oxygen-containing functional groups on the biochar surface, and ion exchange of interlayer anions jointly promoted the adsorption of Cd(II).

The adsorption capacity of LDHs/biochar composites is jointly regulated by the properties of the biochar matrix, the composition of the LDH system, and the synthesis route. A comparative analysis of the data summarized in Table 3 reveals the following patterns:

1. LDH–biochar Synergistic Effect

The adsorption capacities of the LDHs/biochar composite for Pb(II) and Cu(II) are significantly higher than those of pure layered double hydroxides and pristine biochar [66]. This result is consistent with previous findings [30,34], indicating that LDHs/biochar composites not only effectively enhance the removal efficiency of heavy metal ions but also exhibit satisfactory adsorption performance toward organic pollutants and phosphate, demonstrating their broad-spectrum purification potential for multiple types of contaminants.

2. Biomass Feedstock Effect

Even when the LDH system is held constant, the choice of biomass precursor exerts an influence on adsorption performance. For instance, MgAl-LDH composites prepared with coconut shell biochar and pine sawdust biochar as carriers yielded Pb(II) adsorption capacities of 294.0 mg/g [66] and 591.1 mg/g [63], respectively. This nearly twofold difference likely stems from variations in the specific surface area, pore structure, and surface functional group density inherent to the different biomass matrices, as well as the specific preparation conditions employed [97,98].

3. LDH Composition Effect

Conversely, when the biomass feedstock is fixed, the composition of the LDH system also governs adsorption performance. The adsorption capacity of CoAl-LDH/rice husk biochar for Cu(II) exceeds that of ZnMgAl-LDH/rice husk biochar [70,81], underscoring that the structural design of the LDH component must be tailored to the characteristics of the target pollutant for optimal performance.

4. Preparation Method Effect

The hydrothermal, coprecipitation, and ball milling methods each offer distinct advantages for composite synthesis [66,80,82,83]. However, direct comparison across different preparation methods is complicated by multiple confounding factors. Nevertheless, the collective evidence—including the data in Table 1 and the findings of reference [99]—indicates that the coprecipitation method generally yields composites with superior adsorption performance. In addition, this method is operationally simple. Therefore, it is the suitable candidate for industrial scale-up.

In summary, LDHs/biochar composites exhibit broad-spectrum efficacy in removing heavy metal ions from aqueous solutions, with adsorption capacities exceeding those of their individual components. Their performance can be rationally tuned by selecting appropriate biomass feedstocks, optimizing the LDH metal composition, and choosing a suitable synthesis route.

## 5. Conclusions and Future Outlook

The LDHs/biochar composite exhibits a significant synergistic effect, overcoming the limitations of single-layered double hydroxides that are prone to agglomeration and biochar with poor adsorption selectivity. The porous nature and large specific surface area of biochar facilitate the loading of LDHs, which can effectively inhibit the agglomeration of LDH nanoparticles and promote their uniform dispersion, thereby significantly improving the adsorption capacity and adsorption rate of the composite material for both organic and inorganic pollutants.

Meanwhile, this composite material inherits the unique layered structure and adjustable chemical properties of LDHs. Functional design can be achieved by regulating the ratio of metal cations in the layers, the type of interlayer anions, and the modification method of biochar, which enhances the selective removal efficiency for specific target pollutants.

In addition, the precursors of the LDHs/biochar composite are mostly derived from low-cost biomass resources such as agricultural waste and industrial solid waste, and the material itself has regeneration potential, which gives it an advantage in the sustainable utilization of resources. Based on the current research progress, the further development and large-scale application of LDHs/biochar composite materials still need to achieve breakthroughs around three key directions: targeted design, in-depth mechanism research, and recycling.

In terms of material design oriented to target pollutants, it is necessary to accurately construct an active component system of LDHs according to the complex characteristics of multi-pollutant systems. By regulating the type and ratio of metal cations (M^2+^/M^3+^) in the LDH layers and the type of interlayer anions, the selective recognition and efficient removal of specific pollutants can be achieved.

Taking anionic pollutants such as phosphate as an example, the interlayer charge density of LDH systems (e.g., NiAl-LDH and MgAl-LDH) can be optimized. At the same time, considering the structural characteristics and resource advantages of biomass precursors, low-cost biomass such as agricultural waste and industrial waste should be prioritized. By regulating the pyrolysis temperature and modification method, the pore structure and functional group composition of biochar are optimized, and the interfacial bonding between LDHs and biochar is strengthened, forming a composite system with complementary structures, which not only overcomes the agglomeration of LDHs and the poor selectivity of biochar but also improves the selectivity and adsorption capacity of the material.

In terms of mechanism and structure–activity relationship, it is important to elucidate the pollutant removal mechanism, especially the adsorption–degradation pathway of organic pollutants. Advanced characterization techniques and theoretical calculations can be used to systematically analyze the synergistic mechanism of LDHs/biochar toward pollutants, including key processes such as the physical adsorption of biochar, the synergistic effect of chemical adsorption and catalytic degradation of LDHs, interlayer anion exchange, and the generation pathway of reactive oxygen species.

Meanwhile, a clear structure–activity relationship should be established to quantify the correlation between material structure parameters (such as LDH loading, specific surface area of biochar, interface binding strength, and interlayer spacing) and pollutant removal efficiency. The dispersibility of LDH nanosheets can be regulated through exfoliation and reassembly strategies, and the microstructure of the composite material can be optimized to improve adsorption efficiency. This provides theoretical support for the targeted design of composite materials and realizes the transformation from “empirical preparation” to “precision regulation”.

In terms of research on recycling methods, it is necessary to develop efficient, low-cost, and environmentally friendly regeneration technologies to address the problems faced by LDHs/biochar composites in practical applications, such as activity attenuation and secondary pollution. Methods such as chemical regeneration, physical regeneration, and advanced oxidation regeneration can be explored to restore the adsorption sites of the material; at the same time, the regeneration process parameters should be optimized in combination with the structural characteristics of the material. For example, the “memory effect” of LDHs can be used to realize the reconstruction of the interlayer structure and the regeneration of active sites, thereby improving regeneration efficiency and cycling stability.

In addition, a regeneration performance evaluation system should be established to systematically study the evolution of the material’s structure and performance during cyclic use. When combined with life-cycle assessment, the full-chain environmental sustainability of material preparation, application, and regeneration can be achieved, reducing application costs and facilitating the translation of LDHs/biochar composites from laboratory research to large-scale industrial applications.

## Figures and Tables

**Table 2:** Phosphate adsorption capacity of composites under various preparation and adsorption conditions.

No	LPI	BRM	SSA (m^2^/g)	Preparation Condition of Composite	Adsorption Condition	AC (mg/g)	MAC (mg/g)	References
1	CaLa	wheat straw	47.37	*n*(M^2+^):*n*(M^3+^) = 3; pH = 11; Coprecipitation temperature: 60 °C; *t* = 10 h	room temperature; *t* = 10 h; *c*_0_ = 50 mg/L; *m*(adsorbent) = 0.5 g/L	100	166.15 ^1^	[61]
2	MgLa		77.19			29.04	-	
3	CaAl		5.57			76.51	-	
4	CaFe		26.26			62.89	-	
5	MgAl		3.32			15.72	-	
6	MgFe		25.06			17.38	-	
7	ZnFe	rice husk	60	*n*(M^2+^):*n*(M^3+^) = 2; pH = 11; room temperature; *t* = 22 h	temperature: 25 °C; *t* = 1–3 d; *c*_0_: 10–500 mg/L; *m*(adsorbent) = 2 g/L; pH = 6.5	-	27.34	[63]
8	MgFe		105			-	55.59	
9	ZnAl		131			-	80.07	
10	MgAl		148			-	95.90	
11	ZnAl	banana straw	7.02	*n*(M^2+^):*n*(M^3+^) = 10; reacted at 110 °C for 4 h; aged at 60 °C for 8 h	temperature: 20, 30, 40 °C; *t* = 4 h; *c*_0_: 10–600 mg/L; *m*(adsorbent) = 4 g/L	-	178.57 ^2^	[66]
							181.82 ^3^	
							185.19 ^4^	
12	MgAl	corn stalks	26.27	*n*(M^2+^):*n*(M^3+^) = 4; pH = 10–11; reacted at room temperature for 24 h; aged at 65 °C for 24 h; pyrolyzed at 450 °C for 2 h	temperature: 25 °C; *t* = 12 h; *c*_0_: 2.5–500 mg/L; *m*(adsorbent) = 100 g/L	-	54.932	[67]

^1^ The maximum adsorption capacity with initial concentrations of 10–600 mg/L; Adsorption capacity at 20 °C; ^2^ Adsorption capacity at 20 °C; ^3^ Adsorption capacity at 30 °C; ^4^ Adsorption capacity at 40 °C.

**Table 3:** Adsorption Performance of LDHs/biochar Composites with Different Systems for Heavy Metal Ions.

Layered Plate Ions	Biomass Raw Materials	Preparation Method	Target Ions	Adsorption Capacity (mg/g)	pH of the Adsorption Process	Preparation Temperature of Biochar (°C)	References
-	Coconut Shell	Pyrolysis	Pb(II)	13.3	-	900	[36]
MgAl-LDH	-	Coprecipitation	Pb(II)	126.0	-	-	[36]
MgAl-LDH	Coconut Shell	Coprecipitation	Pb(II)	294.0	-	900	[36]
ZnAl-LDH	Coconut Fiber	Coprecipitation	Pb(II)	163.9	6.0	700	[35]
ZnMgAl-LDH	Rice Husk	Coprecipitation	Pb(II)	124.0	6.0	500	[89]
MgAl-LDH	Pinewood Sawdust	Hydrothermal synthesis	Pb(II)	591.2	7.0	350	[90]
MgFe-LDH	Sawdust	Coprecipitation	Pb(II)	335.9	5	500	[91]
-	Coconut Shell	Pyrolysis Method	Cu(II)	11.4	-	900	[36]
MgAl-LDH	-	Coprecipitation	Cu(II)	22.7	-	900	[36]
MgAl-LDH	Coconut Shell	Coprecipitation	Cu(II)	38.6	-	900	[36]
ZnMgAl-LDH	Rice Husk	Coprecipitation	Cu(II)	117.0	6.0	500	[89]
Co-Al LDH	Rice Husk	Mixing	Cu(II)	1377.9	7	500	[92]
MgFe-LDH	Sawdust	Coprecipitation	Cd(II)	209.0	5	500	[91]
MgAl-LDH	Corn Stalk	Ball Milling	Cd(II)	119.0	5.5	-	[93]
CaMgAl-LDH	Bamboo	Hydrothermal synthesis	Cd(II)	216.2	4.5	800	[40]
MgAl-LDH	Pinewood Sawdust	Hydrothermal synthesis	CrO_4_^2−^	330.8	2	350	[90]
Mg/Al- LDH	Praxelis Clematidea	Coprecipitation	Cr(VI)	177.9	2	500	[94]
ZnAl-LDH	Grape Stalks	Coprecipitation	Hg(II)	1280.0	2	350	[95]
CaMgAl-LDH	Bambo	Hydrothermal synthesis	As(III)	206.6	4.5	800	[40]
MgFe-LDH	Pomelo Peel	Coprecipitation	La(III)	42.0	6.0	-	[96]
MgAl-LDH	Bamboo	Coprecipitation	U(VI)	388.8	4	200 ^1^	[38]

^1^ This biochar was prepared by the hydrothermal method at 200 °C, while all others were prepared by the pyrolysis method.

## Data Availability

No new data were created or analyzed in this study. Data sharing is not applicable to this article.
